# Living Light 2018: Conference Report

**DOI:** 10.3390/biomimetics3020011

**Published:** 2018-05-29

**Authors:** Olimpia D. Onelli, Bodo D. Wilts, Silvia Vignolini

**Affiliations:** 1 Melville Laboratory for Polymer Synthesis, Department of Chemistry, University of Cambridge, Lensfield Road, Cambridge CB2 1EW, UK; odo22@cam.ac.uk; 2 Adolphe Merkle Institute, University of Fribourg, Chemin des Verdiers 4, 1700 Fribourg, Switzerland

**Keywords:** living light, biophotonics, biomimetics, biomaterials, evolution, development of nanostructures, structural color, bioluminescence, ecology, biochemistry

## Abstract

Living Light is a biennial conference focused on all aspects of light–matter interaction in biological organisms with a broad, interdisciplinary outlook. The 2018 edition was held at the Møller Centre in Cambridge, UK, from April 11th to April 14th, 2018. Living Light’s main goal is to bring together researchers from different backgrounds (e.g., biologists, physicists and engineers) in order to discuss the current state of the field and sparkle new collaborations and new interdisciplinary projects. With over 90 national and international attendees, the 2018 edition of the conference was strongly multidisciplinary: oral and poster presentations encompassed a wide range of topics ranging from the evolution and development of structural colors in living organisms and their genetic manipulation to the study of fossil photonic structures.

## 1. Introduction

The Living Light Conference Series originated from the idea of forming a forum where biologists, chemists, physicists, and engineers could gather to share their research findings and start multidisciplinary collaborations. In fact, while the interest for biological structures that possess extraordinary optical properties had fast increased since the late 1990s, events bringing together researchers from different sciences in multidisciplinary settings were scarce as every field was firmly based on its own home turf.

In 2009 Prof. Jean-Pol Vigneron organized the first meeting entitled “Workshop on bio-inspired photonic structures” in San Sebastián, the Basque Autonomous Community, Spain. The event was a great success and in 2011 the experience was repeated in Shanghai, China. This meeting, entitled “International symposium on natural photonic structures” was organized by Prof. Jian Zi. The title reflected the increasing number of botanists and entomologists who had become involved in shaping the direction of the field in the recent years. Following the premature loss of Prof. Jean-Pol Vigneron in 2013, it was decided that the conference cycle should be repeated every two years in honor of the pioneering work that he promoted. The first memorial meeting was chaired by Prof. Philippe Lambin at the University of Namur, Belgium in 2014. On this occasion the name “Living Light” was adopted for the first time.

Two years later, Dr. Dimitri Deheyn hosted Living Light at the Scripps Institution of Oceanography in San Diego, CA, USA. This year, the conference was chaired by Dr. Silvia Vignolini in Cambridge, UK.


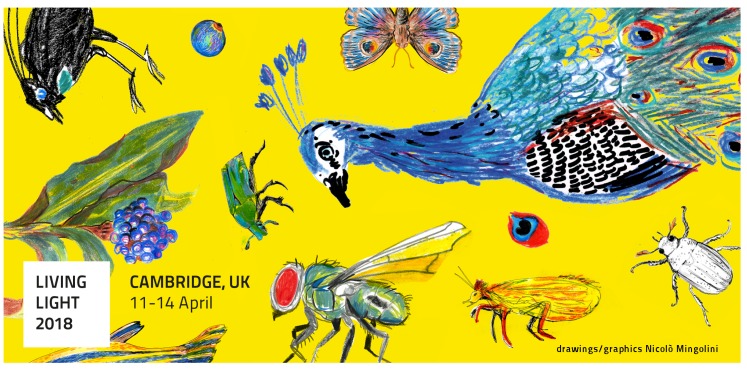
Illustration of the conference themes by Nicolò Mingolini, Living Light’s artist in residence who curated the graphics of the conference materials. The artist’s impressions show some of the colorful organisms discussed at the meeting.

## 2. Sessions

In this conference report, we will summarize the key content and concepts presented at the conference in 44 oral contributions and 21 poster presentations and summarize the main discussion points of two plenary sessions.

To present each presenters work in detail lies outside the scope of this conference report. The event’s program was based on grouping similar topics within the same session, with a keynote speaker introducing the main theme. We here roughly follow this rationale and present the prevalent topics and new ideas that emerged from the conference sessions.

### 2.1. Vision

Prof. N. Justin Marshall opened the 2018 Living Light Conference with an invited talk on stomatopod crustaceans and their highly sensitive vision system [1,2,3]. Vision was a central theme at this year’s meeting with Prof. Nico K. Michiels’ contribution on diurnal photolocation in fish [4] and Prof. Daniel Osorio’s talk on the perception of color [5,6].

Dr. Trevor J. Wardill introduced the audience to the dynamic colors of the sophisticated skin of cephalopods and their polarized intraspecific signals [7]. Dr. Kathryn D. Feller described the discovery of a reflective structure within the photoreceptors of stomatopods [8].

### 2.2. Plant Photonics

Dr. Heather M. Whitney presentation opened the session on plant photonics. The topics discussed included the function of blue leaves and iridescence in *Begonia* sp. as well as a discussion of mechanisms to increase the photosynthetic efficiency of chloroplasts [9]. Nathan J. Masters followed up with a contribution on the dynamic structural color changes in the brown alga *Cystoseira tamariscifolia* [10]. Light management in plants was also discussed in the context of the Goblin’s gold moss by Dr. Martin Lopez-Garcia [11].

Moving from leaves to fruits, Miranda Sinnott-Armstrong’s study of *Viburnum* spp. [12] showed that there is still so much to be explored in the plant world. Rox Middleton showed her results regarding the development of structural color in *Pollia condensata* [13]. Lisa M. Steiner presented a study of the cell wall biochemistry in *Margaritaria nobilis* [14].

### 2.3. Development and Pattern Formation in Nature

Developmental studies gained more attention in this year’s conference with the contributions of Dr. Bodo D. Wilts on gyroid photonic crystals of butterfly wing scales [15], Dr. Olimpia D. Onelli on chitin–melanin multilayers in *Gastrophysa viridula* beetles [16], and Anthony D. McDougal on the development of lepidopteran scales [17,18]. The recent progress towards the understanding of structural color development was greatly facilitated by improvements in imaging techniques. As an example, Prof. Siegfried Reipert described the advantages of a novel cryo-preparation method for a range of biological samples that can be employed for transmission electron microscopy [19].

Pattern formation was also a central topic that keynote speaker Dr. Dvir Gur introduced in the context of zebrafish [20] and was then further explored by other contributed talks such as Rachel C. Thayer’s genetic study of color in *Junonia coenia* butterflies [18] and Dr. Colin J. Ingham’s talk on the manipulation of the genes involved in the structural coloration of *Flavobacterium* IR1 [21]. Dr. Nicola J. Nadeau presented work on crossing iridescent and non-iridescent *Heliconius* butterflies [22], while Jordan Ferria presented his ongoing study of the genetic understanding and manipulation of the surface structures in *Hibiscus trionum* petals [23]. Dr. Jan F. Totz showed the experimental verification of spiral wave chimera states in large oscillator systems, which are likely to occur in living tissues such as cilia carpets [24].

### 2.4. Fossilization of Structural Colors

Dr. Maria E. McNamara opened the session on fossil structural color with an invited talk on the importance of taphonomic experiments where the extreme environmental conditions under which fossils form are replicated in the laboratory [25]. Such decay and maturation studies were also the main topic of Dr. Giliane P. Odin’s presentation on the potential for the conservation of helicoidal multilayers in beetles [26].

Multilayers are the most common type of photonic structures encountered in fossils. However, Dr. Luke T. McDonald’s contribution on the discovery of a three-dimensional color-producing structure in fossil beetles [27] and Dr. Liliana D’Alba’s work on reconstructing the golden appearance of fossil moths shows the potential for other types of structures to be described in the future years [26].

### 2.5. Evolution and Ecology

Dr. Mary Caswell Stoddard opened the session on evolution and ecology with an invited talk exploring various aspects of avian color: from the coevolution between cuckoos and hosts to the invention of a novel computational method to simulate the birds’ visual experience [28,29].

Some of the colors of nature are utterly striking in their conspicuousness. However, often the biological function and evolutionary history are far less evident and require an in-depth study of the ecology and behavior of the organisms involved. For instance, Dr. Bram Vanthournout explored the iridescence of springtails (Collembola) scales: given their limited vision system and the low light conditions of their habitat, it can be hypothesized that their colorful appearance is a side-effect of a nanostructure optimized for thermoregulation.

Prof. Adriana D. Briscoe explored the ecological function of yellow bars in *Heliconius* butterflies, which were revealed to be important in mate selection while not constituting a cost in terms of predator visibility [30]. In contrast, the issue of visibility is a driving factor in the evolution of the transparent butterflies studied by Dr. Doris Gomez and Dr. Marianne Elias [31]. Their study, in fact, revealed that transparency has evolved multiple times through the evolutionary gain of increased camouflage. The speakers also discovered that aposematic butterflies harbor transparent wings and discussed the potential benefit of this seemingly counterintuitive strategy.

Various functions and strategies in the underwater world were also discussed at Living Light 2018. Dr. Johannes W. Goessling explained the mechanism through which diatoms channel light towards their chloroplasts according to the light conditions of their habitat in order to optimize photosynthesis [32,33]. Photosynthetic efficiency is also a key player in the giant clam system presented by Dr. Amanda L. Holt [34]. Here where a layer of iridocytes covers the mantle tissue and converts the intense light from the environment to a lower intensity. This is more compatible with the photosynthetic requirements of the algae which live in symbiosis with the clam.

In the underwater world, we also observe complex physicochemical processes as illustrated by Prof. Daniel E. Morse in his talk “Recent evolution of reflectin metastability enables tunable control of structural color” [35]. Finally, Dr. Maria V. Plyushcheva elucidated the mechanism behind the electronic donor–acceptor coherence in the scale-worm *Lepidonotus squamatus* [36].

### 2.6. Modelling of Light–Matter Interaction

Numerical simulations have traditionally been a cornerstone of the research in biophotonics as they make it possible to correlate the nanostructures observed with the measured optical response as well as predict the optimum parameters for engineering new biomimetic materials. The contributions in this year’s conference highlighted the increased interest in the role of disorder within natural photonic structures. In fact, as Prof. Matthew Shawkey’s talk on the simulation of colors in hummingbird feathers showed, including more parameters in a model can drastically change the results and complicate the interpretation [37,38].

Dr. Bor-Kai Hsiung’s talk “Modulating iridescence in structural colors through hierarchy, micro-geometry, but randomness” showed the importance of disorder in modeling and prototyping bioinspired materials [39,40]. Dr. Andrew Parnell discussed another disordered system, the scales of the white beetle *Cyphochilus* sp. [41,42] and the study of their topology through advanced imaging techniques. Hugo Gruson showed how the irregularities in the patterns of 36 hummingbird species [43,44] affect the optical appearance of the structurally colored feathers. Polydispersity and disorder also play an important role in the squid lens studied by Prof. Alison Sweeney [45] and the Bouligand arcs in Prof. Kenneth Järrendahl’s presentation on chiral reflectors in Scarabidae [46]. Anisotropy was also found in the fluorescence of beetle’s photonic structures by Dr. Sébastien R. Mouchet [47] and Dr. Villads E. Johansen’s work on the arrangement of bacterial colonies that produce structural colors [21].

### 2.7. Biomimetics and Bioinspiration

While the majority of talks focused on the fundamental understanding of organismal structural color, there were also a number of contributions regarding the application of the natural principles for engineering new materials. There was a general increased interest around the theme of dynamic colors and smart materials. For example, Prof. Alon A. Gorodetsky introduced his work on adaptive camouflage materials inspired by cephalopods [48]. Prof. Mathias Kolle talked about the invention of plant-inspired photonic fibers whose color is responsive to stretching and compression [49]. Franziska Schenk discussed her work on iridescent art using novel paints that changes appearance depending on the angle of observation [50,51].

Multifunctional materials were the central theme of Dr. Hendrik Hölscher’s presentation stemming from inspiration by the surfaces of insects that have both optical and self-cleaning functions [52,53]. Two talks were instead concerned with the invention of novel techniques to enhance photosynthetic efficiency: Thomas A. Swift showed the results from the fabrication of a novel method to increase crop productivity using carbon nanodots [54], while Dr. Daniel Wangpraseurt presented a 3D printing-based approach to mimic the nearly ideal quantum efficiency of corals [55]. Also concerned with 3D printing techniques, Prof. Michael Kühl illustrated the potential of using hydrogel scaffoldings as matrices for photosynthesis and respiration [56].

## 3. Poster Contributions

The poster session took place in the panoramic tower of the Møller Centre in a 2 h session preceding the conference dinner at Saint John’s College. The variety in the topics presented in the oral contributions was reflected in the poster presentations. Tescan sponsored the poster prizes which were awarded by the judging committee (Prof. Michael Kühl, Prof. Siegfried Reipert, Dr. Chiara Airoldi, and Franziska Schenk). Pascal Freyer from the University of Groningen (The Netherlands), won the 1st prize with his poster on the structural coloration of blue peacock feathers [57]. Dr. Esteban Bermúdez-Ureña (Adolphe Merkle Institute, Switzerland), received the 2nd prize for his poster on scarab beetle-inspired helicoidal multilayers [58,59]. The 3rd prize went to Lisa M. Steiner (University of Cambridge, UK), for her poster presenting work on “The many (sur)faces of *M. thailandicum*” [60].


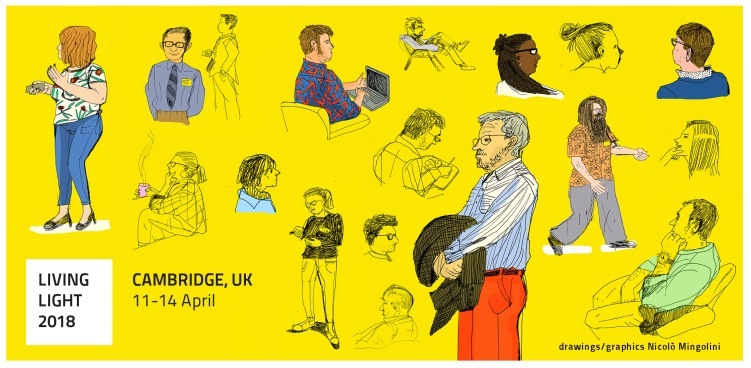
Some of the conference attendees drawn through the eyes of Nicolò Mingolini, Living Light’s artist in residence.

## 4. Plenary Discussions

The 2018 Living Light conference included two plenary discussions chaired by Dr. Silvia Vignolini. The discussions provided a space to spark the debate on common and controversial topics. These constituted also a forum for debating together the future directions of the field and setting the guidelines for the community.

The first session was focused on the tools, relevance, and future of the Living Light community. The panel was constituted by Prof. Daniel E. Morse, Prof. Beverley J. Glover, Prof. Matthew D. Shawkey, and Dr. Bodo D. Wilts. The second session was centered around the themes of biomimetics and bioinspiration. The panel included Prof. Alon A. Gorodetsky, Prof. Mathias Kolle, Prof. Alison M. Sweeney, and Dr. Hendrik Hölscher.

The plenary discussions also benefited from the active participation of the audience and were at times controversial—though never unfriendly—when discussing certain matters, while other topics could be largely agreed upon. Below we give a short overview of the key topics and debate points of each plenary session.

### 4.1. Living Light: Tools, Relevance, and Outlook

- *E-archives*: the panel debated whether the Living Light community would benefit from using e-print services such as arXiv, bioRxiv, vixRa, etc. as repository for unpublished work. Interestingly, the discussion of this topic was rather controversial and no agreement could be reached. While some researchers supported such platforms as an opportunity to shorten publication time and make their work citable early on, others believed that the use of repositories that are not peer-reviewed is dangerous as it can lead to the spread of inaccurate and potentially erroneous studies.
- *Networking and new tools*: the discussion on new tools highlighted that while most hardware (e.g., advanced electron microscopes) and software (e.g., code for numerical simulations) required for the study of biological photonics are already in place, the connection between researchers with different skill sets is still lagging behind. The community agreed that multidisciplinary conferences such as Living Light are crucial to develop new collaborations and broaden the network of scientists working in the field.

### 4.2. The Future of Biomimetics: Scaling Up

- *Developmental studies as a gateway to biomimetics*: there was general consensus that by studying the growth of photonics in living organisms one can gain information useful to mimic the processes in vitro in order to produce low-cost high-performance materials. However, the panel agreed that the complexity of living organisms makes this task extremely challenging and that replicating processes such as DNA synthesis is still far from the capabilities of the current available technologies.
- *Bioinspiration vs. biomimetics*: interestingly, there was no agreement on the use of these two terms and which one of the two processes should be pursued by the Living Light community. While some panelists believed that biomimetics should be the final aim, others were more prone to support bioinspiration as this allows for more freedom in terms of choosing the functions needed for new materials and combine strategies that are not necessarily observed in the same species.
- *Sustainability*: the panel highlighted the need for environmental awareness. Even though biomimetic technologies are inspired by natural structures, they are not necessarily based upon sustainable materials. Therefore the need arises to reflect on the environmental impact of one’s own research with the goal to not only mimic the natural architectures and processes but also to try and questions one’s choice of materials.

## 5. Conclusions

Living Light 2018 was a great success as a result of the high quality of the oral and poster contributions and thanks to the friendly atmosphere which helped strengthen the network of scientists involved in the study of light in living organisms. We are looking forward to the successful continuation of this conference series.

We are pleased to announce that the Living Light Conference Series will continue its biennial cycle and will move to Australia for the 2020 edition. Prof. N. Justin Marshall (Queensland Brain Institute) and Dr. Gerd Schröder-Turk (Murdoch University) will chair the meeting. We look forward to see the community coming together once more to relive the vibrant atmosphere of Living Light.

## References

[1] Daly I.M., How M.J., Partridge J.C., Temple S.E., Marshall N.J., Cronin T.W., Roberts N.W. (2016). Dynamic polarization vision in mantis shrimps. Nat. Commun..

[2] Thoen H.H., Chiou T.H., Marshall N.J. (2017). Intracellular recordings of spectral sensitivities in stomatopods: A comparison across species. Integr. Comp. Biol..

[3] Thoen H.H., Sayre M.E., Marshall J., Strausfeld N.J. (2018). Representation of the stomatopod’s retinal midband in the optic lobes: Putative neural substrates for integrating chromatic, achromatic and polarization information. J. Comp. Neurol..

[4] Michiels N.K., Seeburger V.C., Kalb N., Meadows M.G., Anthes N., Mailli A.A., Jack C.B. (2018). Controlled iris radiance in a diurnal fish looking at prey. R. Soc. Open Sci..

[5] Wilkins L., Marshall N.J., Johnsen S., Osorio D. (2016). Modelling colour constancy in fish: Implications for vision and signalling in water. J. Exp. Biol..

[6] Lind O., Henze M.J., Kelber A., Osorio D. (2017). Coevolution of coloration and colour vision?. Phil. Trans. R. Soc. B.

[7] Kingston A.C., Wardill T.J., Hanlon R.T., Cronin T.W. (2015). An unexpected diversity of photoreceptor classes in the longfin squid, *Doryteuthis pealeii*. PLoS ONE.

[8] Feller K.D., Cronin T.W. (2016). Spectral absorption of visual pigments in stomatopod larval photoreceptors. J. Comp. Phys. A.

[9] Jacobs M., Lopez-Garcia M., Phrathep O.P., Lawson T., Oulton R., Whitney H.M. (2016). Photonic multilayer structure of *Begonia* chloroplasts enhances photosynthetic efficiency. Nat. Plant..

[10] Lopez-Garcia M., Masters N., O’Brien H.E., Lennon J., Atkinson G., Cryan M.J., Oulton R., Whitney H.M. (2018). Light-induced dynamic structural color by intracellular 3D photonic crystals in brown algae. Sci. Adv..

[11] Ignatov M.S., Ignatova E.A., Belousova A.A., Sigaeva A.O. (2012). Additional observations on protonemata of *Schistostega pennata* (Bryophyta). Arctoa.

[12] Gebeshuber I.C., Lee D.W. (2012). Nanostructures for Coloration (Organisms other than Animals).

[13] Vignolini S., Rudall P.J., Rowland A.V., Reed A., Moyroud E., Faden R.B., Baumberg J.J., Glover B.J., Steiner U. (2012). Pointillist structural color in *Pollia* fruit. Proc. Natl. Acad. Sci. USA.

[14] Vignolini S., Gregory T., Kolle M., Lethbridge A., Moyroud E., Steiner U., Glover B.J., Vukusic P., Rudall P.J. (2016). Structural colour from helicoidal cell-wall architecture in fruits of *Margaritaria nobilis*. J. R. Soc. Interface.

[15] Wilts B.D., Zubiri B.A., Klatt M.A., Butz B., Fischer M.G., Kelly S.T., Spiecker E., Steiner U., Schröder-Turk G.E. (2017). Butterfly gyroid nanostructures as a time-frozen glimpse of intracellular membrane development. Sci. Adv..

[16] Onelli O.D., van de Kamp T., Skepper J.N., Powell J., dos Santos Rolo T., Baumbach T., Vignolini S. (2017). Development of structural colour in leaf beetles. Sci. Rep..

[17] Ghiradella H. (1994). Structure of butterfly scales: Patterning in an insect cuticle. Microsc. Res. Tech..

[18] Dinwiddie A., Null R., Pizzano M., Chuong L., Krup A.L., Tan H.E., Patel N.H. (2014). Dynamics of F-actin prefigure the structure of butterfly wing scales. Dev. Biol..

[19] Hollergschwandtner E., Schwaha T., Neumüller J., Kaindl U., Gruber D., Eckhard M., Stöger-Pollach M., Reipert S. (2017). Novel mesostructured inclusions in the epidermal lining of *Artemia franciscana* ovisacs show optical activity. PeerJ.

[20] Gur D., Nicolas J.D., Brumfeld V., Bar-Elli O., Oron D., Levkowitz G. (2018). Development of high-order organization of guanine-based reflectors underlies the dual functionality of the zebrafish iris. bioRxiv.

[21] Johansen V.E., Catón L., Hamidjaja R., Oosterink E., Wilts B.D., Rasmussen T.S., Sherlock M.M., Ingham C.J., Vignolini S. (2018). Genetic manipulation of structural color in bacterial colonies. Proc. Natl. Acad. Sci. USA.

[22] Parnell A.J., Bradford J.E., Curran E.V., Washington A.L., Adams G., Brien M.N., Burg S.L., Morochz C., Fairclough J.P.A., Vukusic P. (2018). Wing scale ultrastructure underlying convergent and divergent iridescent colours in mimetic *Heliconius* butterflies. J. R. Soc. Interface.

[23] Vignolini S., Moyroud E., Hingant T., Banks H., Rudall P.J., Steiner U., Glover B.J. (2015). The flower of *Hibiscus trionum* is both visibly and measurably iridescent. New Phytol..

[24] Totz J.F., Rode J., Tinsley M.R., Showalter K., Engel H. (2018). Spiral wave chimera states in large populations of coupled chemical oscillators. Nat. Phys..

[25] McNamara M., Field D. (2016). Maturation Experiments Reveal Bias in the Fossil Record of Feathers. Proceedings of the EGU General Assembly Conference Abstracts.

[26] Zhang Q., Mey W., Ansorge J., Starkey T.A., McDonald L.T., McNamara M.E., Jarzembowski E.A., Wichard W., Kelly R., Ren X. (2018). Fossil scales illuminate the early evolution of lepidopterans and structural colors. Sci. Adv..

[27] McNamara M.E., Saranathan V., Locatelli E.R., Noh H., Briggs D.E., Orr P.J., Cao H. (2014). Cryptic iridescence in a fossil weevil generated by single diamond photonic crystals. J. R. Soc. Interface.

[28] Stoddard M.C., Kupán K., Eyster H.N., Rojas-Abreu W., Cruz-López M., Serrano-Meneses M.A., Küpper C. (2016). Camouflage and clutch survival in plovers and terns. Sci. Rep..

[29] Stoddard M.C., Hauber M.E. (2017). Colour, vision and coevolution in avian brood parasitism. Philos. Trans. R. Soc. B.

[30] Finkbeiner S.D., Fishman D.A., Osorio D., Briscoe A.D. (2017). Ultraviolet and yellow reflectance but not fluorescence is important for visual discrimination of conspecifics by *Heliconius erato*. J. Exp. Biol..

[31] Willmott K.R., Willmott J.C.R., Elias M., Jiggins C.D. (2017). Maintaining mimicry diversity: Optimal warning colour patterns differ among microhabitats in Amazonian clearwing butterflies. Proc. R. Soc. B.

[32] Goessling J.W., Frankenbach S., Ribeiro L., Serôdio J., Kühl M. (2018). Modulation of the light field related to valve optical properties of raphid diatoms: Implications for niche differentiation in the microphytobenthos. Mar. Ecol. Prog. Ser..

[33] Goessling J.W., Su Y., Cartaxana P., Maibohm C., Rickelt L.F., Trampe E.C., Walby S.L., Wangpraseurt D., Wu X., Ellegaard M. (2018). Structure-based optics of centric diatom frustules: Modulation of the in vivo light field for efficient diatom photosynthesis. New Phytol..

[34] Holt A.L., Vahidinia S., Gagnon Y.L., Morse D.E., Sweeney A.M. (2014). Photosymbiotic giant clams are transformers of solar flux. J. R. Soc. Interface.

[35] Levenson R., DeMartini D.G., Morse D.E. (2017). Molecular mechanism of reflectin’s tunable biophotonic control: Opportunities and limitations for new optoelectronics. APL Mater..

[36] Plyushcheva M.V., da Silva K.P., Aneli N.B., Vays V.B., Kondrashov F.A., Goñi A.R. (2017). Two-color fluorescence in elytra of the scale-worm *Lepidonotus squamatus* (Polychaeta, Polynoidae): In vivo spectral characteristic. Mater. Today Proc..

[37] Xiao M., Hu Z., Wang Z., Li Y., Tormo A.D., Le Thomas N., Wang B., Gianneschi N.C., Shawkey M.D., Dhinojwala A. (2017). Bioinspired bright noniridescent photonic melanin supraballs. Sci. Adv..

[38] Peteya J.A., Clarke J.A., Li Q., Gao K.Q., Shawkey M.D. (2017). The plumage and colouration of an enantiornithine bird from the Early Cretaceous of China. Palaeontology.

[39] Hsiung B.K., Siddique R.H., Jiang L., Liu Y., Lu Y., Shawkey M.D., Blackledge T.A. (2017). Tarantula-inspired noniridescent photonics with long-range order. Adv. Opt. Mater..

[40] Hsiung B.K., Siddique R.H., Stavenga D.G., Otto J.C., Allen M.C., Liu Y., Lu Y.F., Deheyn D.D., Shawkey M.D., Blackledge T.A. (2017). Rainbow peacock spiders inspire miniature super-iridescent optics. Nat. Commun..

[41] Vukusic P., Hallam B., Noyes J. (2007). Brilliant whiteness in ultrathin beetle scales. Science.

[42] Burresi M., Cortese L., Pattelli L., Kolle M., Vukusic P., Wiersma D.S., Steiner U., Vignolini S. (2014). Bright-white beetle scales optimise multiple scattering of light. Sci. Rep..

[43] Greenewalt C.H., Brandt W., Friel D.D. (1960). Iridescent colors of hummingbird feathers. J. Opt. Soc. Am..

[44] Durrer H. (1986). Colouration. Biology of the Integument.

[45] Cai J., Townsend J., Dodson T., Heiney P., Sweeney A. (2017). Eye patches: Protein assembly of index-gradient squid lenses. Science.

[46] Mendoza-Galván A., Muñoz-Pineda E., Ribeiro S.J., Santos M.V., Järrendahl K., Arwin H. (2018). Mueller matrix spectroscopic ellipsometry study of chiral nanocrystalline cellulose films. J. Opt..

[47] Mouchet S.R., Lobet M., Kolaric B., Kaczmarek A.M., Van Deun R., Vukusic P., Deparis O., Van Hooijdonk E. (2016). Controlled fluorescence in a beetle’s photonic structure and its sensitivity to environmentally induced changes. Proc. R. Soc. B.

[48] Ordinario D.D., Leung E.M., Phan L., Kautz R., Lee W.K., Naeim M., Kerr J.P., Aquino M.J., Sheehan P.E., Gorodetsky A.A. (2017). Protochromic devices from a cephalopod structural protein. Adv. Opt. Mater..

[49] Aizenberg J., Kolle M., Vukusic P., Howe R.D. (2015). Band-Gap Tunable Elastic Optical Multilayer Fibers. U.S. Patent.

[50] Schenk F., Wilts B.D., Stavenga D.G. (2013). The Japanese jewel beetle: A painter’s challenge. Bioinspir. Biomim..

[51] Schenk F., Lakhtakia A., Knez M., Martín-Palma R.J. (2015). Biomimetics, color, and the arts. Bioinspiration, Biomimetics, and Bioreplication 2015.

[52] Siddique R.H., Donie Y.J., Gomard G., Yalamanchili S., Merdzhanova T., Lemmer U., Hölscher H. (2017). Bioinspired phase-separated disordered nanostructures for thin photovoltaic absorbers. Sci. Adv..

[53] Syurik J., Siddique R.H., Dollmann A., Gomard G., Schneider M., Worgull M., Wiegand G., Hölscher H. (2017). Bio-inspired, large scale, highly-scattering films for nanoparticle-alternative white surfaces. Sci. Rep..

[54] Hill S.A., Benito-Alifonso D., Morgan D.J., Davis S.A., Berry M., Galan M.C. (2016). Three-minute synthesis of sp^3^ nanocrystalline carbon dots as non-toxic fluorescent platforms for intracellular delivery. Nanoscale.

[55] Wangpraseurt D., Holm J.B., Larkum A.W., Pernice M., Ralph P.J., Suggett D.J., Kühl M. (2017). In vivo microscale measurements of light and photosynthesis during coral bleaching: Evidence for the optical feedback loop?. Front. Microbiol..

[56] Koren K., Jakobsen S.L., Kühl M. (2016). In-vivo imaging of O_2_ dynamics on coral surfaces spray-painted with sensor nanoparticles. Sens. Actuator B Chem..

[57] Zi J., Yu X., Li Y., Hu X., Xu C., Wang X., Liu X., Fu R. (2003). Coloration strategies in peacock feathers. Proc. Natl. Acad. Sci. USA.

[58] Schmidt O.G., Eberl K. (2001). Nanotechnology: Thin solid films roll up into nanotubes. Nature.

[59] Sharma V., Crne M., Park J.O., Srinivasarao M. (2009). Structural origin of circularly polarized iridescence in jeweled beetles. Science.

[60] Gould K.S., Lee D.W. (1996). Physical and ultrastructural basis of blue leaf iridescence in four Malaysian understory plants. Am. J. Bot..

